# The effect of effort-reward imbalance on the health of childcare workers in Hamburg: a longitudinal study

**DOI:** 10.1186/s12995-017-0163-8

**Published:** 2017-06-26

**Authors:** Peter Koch, Jan Felix Kersten, Johanna Stranzinger, Albert Nienhaus

**Affiliations:** 1 Centre of Excellence for Epidemiology and Health Services Research for Healthcare Professionals (CVcare), University Medical Centre Hamburg-Eppendorf, Martinistrasse 52, 20246 Hamburg, Germany; 2 Health Protection Division (FBG), Institution for Statutory Accident Insurance and Prevention in the Health and Welfare Services (BGW), Pappelallee 33, 22089 Hamburg, Germany

**Keywords:** Musculoskeletal symptoms, Burnout, Psychosocial, Nursery teacher, Occupational disease, Esteem, Work-related

## Abstract

**Background:**

The prevalence of effort-reward imbalance (ERI) among qualified childcare workers in Germany is currently estimated at around 65%. High rates of burnout and musculoskeletal symptoms (MS) have also been reported for this group. Previous longitudinal studies show inconsistent results with regard to the association between ERI and MS. As yet, no longitudinal studies have been conducted to investigate the association between ERI and burnout or MS in childcare workers. This study aims to investigate the extent to which a relationship between ERI and MS or burnout can be observed in childcare workers in Germany on a longitudinal basis.

**Methods:**

In 2014 childcare workers (*N* = 199, response rate: 57%) of a provider of facilities for children and youth in Hamburg were asked about stress and health effects in the workplace. Follow-up was completed one year later (*N* = 106, follow-up rate: 53%) For the baseline assessment, ERI was determined as the primary influencing factor. Data on MS was recorded using the Nordic questionnaire, and burnout using the personal burnout scale of the Copenhagen Burnout Inventory (CBI). The statistical analysis was carried out using multivariate linear and logistic regression.

**Results:**

At baseline ERI was present in 65% of the sample population. The mean burnout score at the time of follow-up was 53.7 (SD: 20.7); the prevalence of MS was between 19% and 62%. ERI was identified as a statistically significant factor for MS, after adjusting especially for physical stress (lower back: OR 4.2; 95% CI: 1.14 to 15.50, neck: OR 4.3; 95% CI: 1.25 to 15.0, total MS: OR 4.0; 95% CI: 1.20 to 13.49). With regard to burnout, a relative increase of 10% in the ERI ratio score increased the burnout score by 1.1 points (*p* = 0.034).

**Conclusions:**

ERI was revealed to be a major factor in relation to MS and burnout in childcare workers. Based on this observation worksite interventions on the individual and organizational level should be introduced in order to prevent ERI.

**Electronic supplementary material:**

The online version of this article (doi:10.1186/s12995-017-0163-8) contains supplementary material, which is available to authorized users.

## Background

Current German studies report unfavourable psychosocial working conditions for childcare workers. According to these studies, the prevalence of work-related effort-reward imbalance [1] is in between 64% and 67% [2–4]. In Siegrist’s effort-reward imbalance model (ERI model), the health of the employee is associated with performance and rewards (esteem, job security and promotion). The model is based on the assumption that there should ideally be a reciprocal relationship between efforts and socially defined rewards. If rewards are lower than efforts, a stressful situation that increases the risk of stress-related diseases occurs for the employee. Empirical evidence for this hypothesis has been found mainly for coronary heart disease, cardiovascular disease and depression [5]. A special feature of the ERI model is the inclusion of over-commitment (OVC) personality as a personal trait that represents a coping strategy in combination with high demands. OVC generates excessive commitment in conjunction with expectations of high rewards. According to Siegrist employees with OVC are also at increased risk, and in combination with ERI even higher risk, for developing stress-related diseases. Observations in German teachers found that OVC negatively affected plasma coagulation, natural killer cells and T-helper cells [6, 7]. Furthermore, depression and somatic symptoms including MS were found to be associated with the interaction of OVC and ERI in nurses [8, 9].

International studies have observed an increased risk of musculoskeletal disorders among childcare workers [10–13]. The association between the increase of MS and the factors of the ERI model has been observed in longitudinal studies of employee cohorts in different industries [14–16]. In a systematic review of all industries, however, the association between ERI and MS has been evaluated as inconsistent on the basis of cross-sectional and longitudinal studies [17]. To our knowledge, there have not yet been performed any longitudinal studies examining the association between ERI and MS in childcare workers.

Another symptom associated with stress in the workplace is burnout. Employees working in the service sector show a high risk of burnout [18]. Childcare workers as an occupational group do not represent any exception to this in international comparisons [19–23]. For childcare workers in Germany, prevalence rates of between 10% and 57% have been observed for burnout symptoms [2, 10, 24, 25]. For childcare workers and teaching staff, ERI shows a strong correlation with burnout [26]. A greater tendency towards OVC was shown to be associated with burnout in cross-sectional studies of qualified childcare workers and across industries [2, 27]. Longitudinal studies investigating the association between ERI and burnout in childcare workers have not been published yet.

We aim to address the following research questions in this study:

1. Does a longitudinal approach reveal an association between the psychosocial factors of the ERI model and MS among childcare workers?
2. Does a longitudinal approach reveal an association between the ERI ratio score and a higher risk of burnout among childcare workers?

## Methods

As part of a 2014 occupational risk assessment a funding provider for children and young people comprising 26 different facilities in Hamburg carried out a stress monitoring survey of its childcare workers [2]. In this paper the results of the follow-up investigation of this multicentre study are presented.

In November 2014, all 400 qualified childcare workers of all different facilities were asked about health and stresses they faced at work. A total of 230 questionnaires were returned (response rate: 57%); a total of 31 participants were excluded as a result of low weekly working hours (< 10 h) and employment in domestic/janitorial services (kitchens, workshops). At the time of the baseline assessment, 199 people were therefore included into the study. After twelve months (follow up), all study participants once again received a copy of the same pseudonymised questionnaire they had completed a year before. A subgroup of participants (*n* = 33) took part in a parallel intervention programme looking at the effects of noise in the workplace [28]. In that study, the focus was on the question of whether the use of personal hearing protection over the observational period of one year could reduce the subjective noise exposure and the risk of burnout among childcare workers.

The pseudonymised stress monitoring questionnaire was agreed with the data safety officer of the funding provider for children and young people. Before the study started, every participant gave informed written consent for taking part in the study. All study documents, including the study protocol, were reviewed and approved by the Hamburg Medical Chamber Ethics Committee as part of an application process (reference: PV4792).

### Questionnaire

In addition to demographic variables, the questionnaire also collected information on work-related stress and resources. Burnout and MS were used as outcomes.

*Physical stress* was recorded using selected questions from a standardised questionnaire [29]. Five different types of stress (*awkward body postures, standing, sitting, lifting heavy loads/children* and *carrying heavy loads/children*) were identified on a four-stage frequency scale. This resulted in a corresponding total score (ranging between 5 and 20). Using the median, the variable was dichotomised into the categories of low or high physical stress.

*Subjective noise exposure* was estimated using a questionnaire developed by the authors. Responding to 13 items on a five-stage scale resulted in a total score (ranging between 13 and 65). This was dichotomised into high and low subjective noise exposure by using the median. For more information, please see the publication of the cross-sectional study [2].

*Psychosocial factors* were recorded using the ERI questionnaire (23-item version) [30]. The psychosocial situation and the personality trait of *OVC* were evaluated using three scales (*effort*: six items, *reward*: eleven items and *OVC*: six items). The ERI ratio score was determined according to the definition using a formula that takes into account the different numbers of items in order to calculate the total on the *effort* scale as a ratio to the *reward* scale: ∑ Effort/∑Reward*0.5454. An effort-reward imbalance was defined as an ERI ratio score of more than 1. Since this value is not a clinically valid cut-off value, ERI was also tested using the quartile thresholds as an ordinal influencing variable in the analysis. Regardless of the scale, increased *OVC* was defined for the value range in the upper tertile of the empirical distribution and treated as a dichotomous variable.

Other workplace-related characteristics were recorded using selected scales from a standardized instrument, the brief workplace analysis questionnaire (KFZA) [31]. This included both stress factors (*qualitative workload: two items, quantitative workload: two items*) and resources (*control: three items, collaboration: three items, information and employee participation: two items, completeness: two items*, *variety: three items).* The individual items were rated on a five-stage scale.

In addition, the respondent was asked about the occurrence of typical everyday situations in the workplace. Seven different statements, such as “I experience conflicts with parents” or “I don’t get any breaks or chances to step away from work for a while” could be answered with yes or no.

*Musculoskeletal symptoms* were recorded using the Nordic questionnaire [32]. The prevalence of chronic pain in the shoulder, neck or lower back was defined as the presence of pain on at least eight days in the past twelve months, as well as pain within seven days of filling in the questionnaire. In addition, a comprehensive variable was derived for the presence of at least one type of chronic pain in the three body regions (MS total).

In order to evaluate *burnout* in childcare workers, the *personal burnout* sub-scale from the Copenhagen Burnout Inventory was used [33]. According to the definition, a higher risk of burnout is present with a value of ≥50 (range 0–100).

### Statistical analysis

For paired group comparisons, the paired t-test was calculated in the case of normally distributed data; for not normally distributed data the Mann–Whitney *U* test was calculated. For dichotomous paired data, the McNemar test was used. For independent data, the Pearson correlation coefficient was used. In order to evaluate a difference in nominal variables, the chi-squared test was used.

Multivariate logistic regression was calculated for the first research question. Starting with a core variable set (ERI, physical stress, pain T0, participation in intervention programme), all variables with a *p*-value of <0.25 in the bivariate analysis were successively integrated into the model [34]. Physical stress was included as an important confounder in the relationship between ERI and MS [16]. The following variables were taken into account as potentially influential variables: *work-related resources and stress (KFZA), typical everyday situations in the workplace, subjective noise exposure, physical stress, weekly working hours, type of institution, field of work, physical activity, age, BMI* and *gender.*

With regard to the second hypothesis, linear regression was used. Starting with a core variable set (ERI, burnout T0, age, participation in a prevention programme, type of institution) all other variables were included that showed a *p*-value of <0.2 in the bivariate analysis. In the second step, the stepwise backwards regression procedure was applied [34], where all variables with *p*-value of >0.1 were excluded from the model. In order to fulfil the requirements of linear regression, the ERI variable was transformed to the logarithmic scaling.

In all multivariate analyses a possible interaction between ERI and OVC was also tested. For logistic regression models a variable with four categories has been built: 1: ERI No/ OVC No, 2: ERI Yes/ OVC No, 3: ERI No/ OVC Yes, 4: ERI Yes/OVC Yes. For linear regression models a multiplicative term has been built from the continuous OVC variable and ERI ratio variable [35].

Missing values were replaced in the ERI scale (effort, reward, OVC) and in the personal burnout scale by individual mean values. If more than half of the individual items on a particular scale were missing for a participant, the entire scale value was set to a missing value.

A dropout analysis was performed using logistic regression. The statistical analysis was carried out using SPSS Statistics, version 23.

## Results

At the time of the follow-up, the cohort comprises 106 employees (see Table 1) (Follow-up rate: 53%). The study participants are predominantly women (90.6%). The study participants in the follow-up are statistically significantly older than the dropouts (43 vs 37, *p* < 0.001); age was the only statistically significant variable in the dropout analysis. More than 90% have German nationality. Almost half of the participants have a BMI of ≥25 (47%). Overall, 51.9% of the employees report regular physical exercise. More than half (52.8%) work full time, with the majority working exclusively in child care (84.9%). Of all of the employees, 66% are from child day care centres, 21.7% work in school partnerships (caring for school-age children in schools) and the lowest proportion (11.3%) come from child and youth support facilities (youth projects and residential groups). As a result of too many missing values (> 50%), working hours are not evaluated.

**Table 1:** Description of the cohort at the time of follow up

Variable	n	Percent
Gender		
Women	96	90.6%
Men	10	9.4%
Age in years		
18–29	16	15.1%
30–39	22	20.8%
40–49	38	35.8%
50+	29	27.4%
n/a	1	0.9%
Nationality		
German	98	92.5%
Other	8	7.5%
BMI		
< 25	54	50.9%
≥ 25	50	47.1%
n/a	2	1.9%
Physical exercise		
Regular	55	51.9%
None	51	48.1%
Area of work		
Child care	90	84.9%
Management/administration	16	15.1%
Weekly working hours		
Full time	56	52.8%
Part time	50	47.2%
Institution		
Child day care centre	70	66%
School partnership	23	21.7%
Child and youth support work	12	11.3%
n/a	1	0.9%
Total	106	100%

**Fig. 1:**
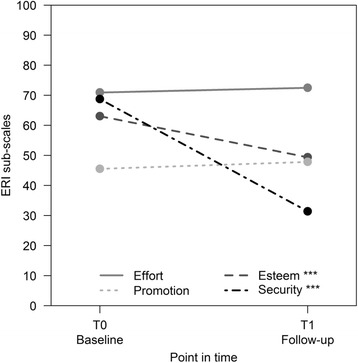
Mean values for the ERI sub-scales on a standardised scale of 0 to 100 (***p < 0.001)

Table 2 shows the influential and outcome variables at the time of baseline and follow-up. In terms of resources, the mean values of the variables are ranging between 3.5 and 3.9 at both points in time. This corresponds to an occurrence of 70–78% in the upper end of the scale for individual resources. The mean value for *collaboration* shows a statistically significant decrease over time. Here, the mean decreases from 3.7 to 3.5 (*p* = 0.006). Among the stress factors, there are no statistically significant changes over time for any variables with the exception of ERI. The ERI ratio score increases from 1.2 to 1.3 points (*p* < 0.001), while the difference in the dichotomised ERI variable is also statistically significant (65.1% vs 87.4%, *p* < 0.001). Figure 1 shows which of the ERI sub-scales is mainly responsible for the significant increase in the ERI ratio score. The mean of the *effort* scale remains nearly constant over time (73 vs. 72). For the three sub-scales of the reward scale, the following trends can be observed: *promotion* increases by three points over time (45 vs 48), *esteem* and *security*, however, decrease statistically significant over time. Here, the mean values decrease from 62 to 49 (*p* < 0.001) and from 67 to 31 (*p* < 0.001) respectively.

**Table 2:** Resources and stress variables and outcomes at the time of baseline and follow-up (*n* = 106)

	Baseline				Follow Up				
Resources	x̅	SD	%	n	x̅	SD	%	n	p
Control (scale: 1–5)	3.7	0.9	.	.	3.6	0.9	.	.	0.228
Variety (scale: 1–5)	3.9	0.7	.	.	3.9	0.6	.	.	0.732
Completeness (scale: 1–5)	3.7	0.8	.	.	3.5	0.9	.	.	0.057
Collaboration (scale: 1–5)	3.7	0.7	.	.	3.5	0.8	.	.	0.006*
Information and employee participation (scale: 1–5)	3.8	0.7	.	.	3.7	0.8	.	.	0.120
Stress factors									
Qualitative workload (scale: 1–5)	2.4	0.8	.	.	2.4	0.8	.	.	0.901
Physical stress (scale: 5–20)	14.3	2.8	.	.	14.3	2.8	.	.	0.792
Subjective noise exposure (scale: 13–65)	39.7	10.3	.	.	40.6	10.5	.	.	0.220
ERI ratio score (scale: 0.2–5)	1.2	0.4	.	.	1.3	0.3	.	.	< 0.001**
ERI > 1, proportion (n)	.	.	65.1	69	.	.	87.4	90	< 0.001**
OVC (scale: 6–24)	15.8	3.4	.	.	15.3	3.5	.	.	0.084
Outcomes									
Burnout (scale 0–100)	50.6	19.7	.	.	53.7	20.7	.	.	0.056
Risk of burnout >50,	.	.	53.8	57	.	.	61.3	65	0.096
Neck pain	.	.	32.1	34	.	.	39.4	41	0.286
Shoulder pain	.	.	15.1	16	.	.	19.2	20	0.523
Lower back pain	.	.	39.6	42	.	.	34.6	36	0.523
MS total	.	.	55.7	59	.	.	62.1	64	0.700

x̅: mean, SD: standard deviation, *p < 0.05, **p < 0.001

With regard to the outcome variables (Table 2), a slight increase in burnout can be observed (50.6 vs 53.7), which is only just not statistically significant (*p* = 0.056). For neck pain (32.1% vs 39.4%), shoulder pain (15.1% vs 19.2%) and MS overall (55.7% vs 62.1%), slight increases can be observed. The prevalence of lower back pain (39.6% vs 34.6%) decreases slightly over time. These differences are not statistically significant.

The results of the multivariate logistic regression of the association between ERI and MS are listed in Table 3. For the outcome of lower back pain, the odds ratio is 4.2 times higher for child care workers with an ERI of >1 (95% CI: 1.14 to 15.50). This correlation is statistically significant. For shoulder pain, an ERI of >1 reveals an increased odds ratio of 1.5 (95% CI: 0.40 to 5.58), which is not statistically significant. In addition, participants with low control show an odds ratio that is 4.5 times higher for shoulder pain (95% CI: 1.15 to 17.42), which is statistically significant. OVC was observed to have a protective effect that was not statistically significant (OR: 0.4; 95% CI: 0.09 to 1.40). With regard to neck pain, an ERI of >1 resulted in a statistically significant higher odds ratio of 4.3 (95% CI: 1.25 to 15.0). For the outcome of total MS, employees with an increased ERI ratio score were also observed to have a statistically significant increase in the odds ratio (OR: 4.0; 95% CI: 1.20 to 13.49). Child care workers who state that they have physical exercise regularly are shown to have a statistically significant protective effect with regard to MS (OR: 0.3; 95% CI: 0.10 to 0.98). Employees who state that they experience conflicts with parents have a statistically significant increase in the risk of MS (OR: 4.9; 95% CI: 1.55 to 15.75). No interaction between ERI and OVC was observed in any of the models.

**Table 3:** Results of the multivariate logistic regressions for development of musculoskeletal symptoms (adjusted for age, MS T0)

Outcome at Follow Up:	Lower back 36 (35%)			Shoulder 20 (19%)			Neck 41 (39%)			MS total 64 (62%)		
	OR	95% CI	p	OR	95% CI	p	OR	95% CI	p	OR	95% CI	p
Influencing variables at baseline:												
ERI >1 vs ≤1	4.2*	1.14–15.50	0.031	1.5	0.40–5.58	0.547	4.3*	1.25–15.00	0.021	4.0*	1.20–13.49	0.024
Physical stress high vs low	2.8	0.94–8.10	0.064	1.1	0.33–3.69	0.876	0.9	0.33–2.61	0.891	1.2	0.37–3.87	0.758
Intervention yes vs. no	0.6	0.19–1.86	0.375	2.0	0.63–6.23	0.245	0.9	0.30–2.56	0.804	0.5	0.13–1.63	0.212
OVC 3rd tertile vs 1st + 2nd tertile	.	.	.	0.4	0.09–1.40	0.138	.	.	.	.	.	.
Control high vs low	.	.	.	4.5*	1.15–17.42	0.031	.	.	.	2.0	0.61–6.69	0.254
Physical activity yes vs no	.	.	.	.	.	.	.	.	.	0.3*	0.10–0.98	0.046
Conflicts with parents yes vs no	.	.	.	.	.	.	.	.	.	4.9*	1.55–15.75	0.007
R^2^	R^2^ = 0.44			R^2^ = 0.19			R^2^ = 0.39			R^2^ = 0.45		

*p < 0.05

With regard to burnout, it is shown that the ERI ratio score has a statistically significant influence on increasing the risk of burnout. Translated to the delogrithmed scaling, an increase in the ERI ratio score of 10% would increase the burnout value by 1.1 points (95% CI: 0.09 to 2.14) (Table 4). This increase is statistically significant (*p* = 0.034). The resource of variety is shown to be a protective factor (beta: –3.8; 95% CI: –0.8 to 0.37), but is not statistically significant. Age reduces the burnout value by 0.6 points per year (95% CI: –0.87 to −0.29), a statistically significant effect (*p* < 0.001). Participation in the intervention programme has a slightly reductive effect on the target variable (beta: –2.4; 95% CI: –8.66 to 3.78). This effect is not statistically significant. In addition, it can also be observed that the burnout value for employees from child day care centres is 7 points higher than for employees from the two other types of institution (95% CI: 0.56 to 13.51). This increase is statistically significant (*p* = 0.034).

**Table 4:** Multivariate linear model for burnout (adjusted for burnout T0)

R^2^: 0.53	Regression coefficient	Standardised beta coefficient	95% CI	p
Increase in the ERI ratio score by 10%	1.1*	0.18	0.09–2.14	0.034
Variety (scale 1–5)	–3.8	−0.14	−8.0 – 0.37	0.074
Intervention yes vs no	−2.4	−0.06	−8.66 – 3.78	0.439
Age (per year)	−0.6*	−0.29	−0.87 – –0.29	0.001
Child day care centres yes vs other institutions	7.0*	0.16	0.56–13.51	0.034

*p < 0.05

**p < 0.001

## Discussion

In this longitudinal study, statistically significant associations between an increased ERI ratio score and the increase of MS were observed in childcare workers. In these analyses physical stress was included as a confounder variable. With regard to increasing the risk of burnout, ERI was also shown to be a statistically significant factor.

### Effort-reward imbalance

We found a high prevalence of ERI among childcare workers (follow-up: 87.4% with ERI ratio > 1; mean ERI ratio: 1.3) in this study, compared to the cross-sectional study from the previous year with a prevalence rate of 65% and a mean ERI ratio of 1.17 [2].

Such unusually high levels of ERI are rare in literature. In an older study investigating childcare workers in 2004, the mean ERI was 0.5 [26]. More recent data assessed in 2012 showed ERI prevalence rates of between 64% and 67% for childcare workers, while for management staff rates of 87% [3, 4]. As was already discussed in the cross-sectional study [2], the increase in ERI over time could potentially be explained by increasing dissatisfaction with working conditions among childcare workers: since 2013, parents in Germany have had a legal right to a childcare place for infants aged 1 to <3, additionally to the existing claim for children aged 3 to 6. In recent years, this has led to larger group sizes, unfavourable staffing conditions and an increase in temporary working contracts. As a result, there was a wave of strikes instigated by childcare workers in Germany in 2015. The questionnaires were returned just a few months after the strikes had taken place. This professional-policy environment is linked with increased awareness of the lack of value accorded to this occupational group in Germany, which is made clear by the drop in the ERI sub-scale *esteem*. The decline in the *job security* sub-scale is also very clear. Paradoxically, almost all staff in the institutions had permanent contracts at the time. The decrease in the two-item *job security* sub-scale was caused in detail by the low scores for the item: “My own job is at risk”. Discussion with employee representatives and the management revealed, that at the time of the follow-up the majority of employees were subject to an internal rotation process in their job. This principle meant that, at that time, employees often switched jobs within an institution or between institutions. In this context, answers to this question on the ERI questionnaire were bound to have been biased.

The prevention of ERI by using an ERI model based worksite stress management program, as demonstrated in interventional studies, is feasible and can positively influence psychosocial work environment and mental health [36, 37]. Aiming to reduce overcommitted work-related attitudes, Aust et al. conducted successfully interventions that were performed on individual and organisational levels [36]. With a participative approach Bourbonnais et al. involved employees of a hospital in formulating goals in terms of psychological demands and rewards. After 12 months a reduction of adverse psychological factors was investigated in the experimental group [37]*.*

### Musculoskeletal symptoms

We found significant associations between ERI and MS in three out of four body regions in qualified childcare workers: back, neck and combination of back, neck and shoulder (MS total). The association between ERI and lower back pain (OR: 4.2) has been observed in other longitudinal studies investigating employees of a transport company [14], employees in public administration [15] and in other cross-sectional studies investigating employees in healthcare, the wine-growing industry, the police and public transport companies [38–41].

There was a tendency towards association between ERI and the increase of shoulder pain in this study (OR: 1.5) but not to a statistically significant degree. Lower control (OR: 4.5) was revealed to be a significant influencing factor with regard to shoulder pain. Control as a psychosocial factor derives from the demand-control-support model [42], another stress model that describes the onset of work-related stress.

With regards to neck pain the ERI variable showed a significant effect (OR: 4.3). This effect was also observed in drivers and office workers as well as in cohorts of hospital staff and workers in the wine-growing industry in two longitudinal studies [14, 15] and three cross-sectional studies [38, 39, 43].

For the outcome of total MS, an increased risk was observed for participants with an ERI >1 (OR: 4.0).

Additionally, two other variables seemed to have had an influence on total MS: perceived conflicts with parents (OR: 4.9) and regular physical exercise (OR: 0.3) as a protective factor showed significant associations with the outcome. Childcare workers could be adequately supported with on-the-job training in conflict management to possibly prevent the increase of MS. Conflict management and company-facilitated sports activities for employees would not only directly influence the onset of MS, but would also indirectly affect ERI: childcare workers might perceive this as a kind of esteem for their seniority.

Indications of an interaction between ERI and OVC were not observed in relation to MS. To our knowledge, there is only one cross-sectional study where an interactive effect of this kind was documented with regard to MS in nursing staff [9].

Regarding the biological plausibility there are several explanations for the mechanism of psychosocial factors leading to MS: psychosocial stress might induce increased and prolonged muscle tension [44] and decreased blood supply in extremities [45]. It also blocks anabolic activity which is responsible for the repair of muscle tissue [46]. Another short-term stress response is muscle violation due to increased sensitivity of muscle fibres [44]. Due to these permanent short term responses the risk of chronic MS might increase over time.

### Burnout

The prevalence of burnout at the time of the follow-up was higher, at 61.3% (mean: 53.7), than in the cross-sectional study one year before (56.8%, mean: 51.7) [2]. The reference data from the COPSOQ database from 2013 shows a mean burnout score for childcare workers of 48 (Additional file 1, Nuebling). The results of the linear regression showed a significant increase in burnout with an increase in ERI ratio (if the ERI ratio increases by 10%, the burnout risk increases by 1.12 points). In a longitudinal study, Spence et al. [47] also observed a significant association between ERI and burnout in nurse managers. Other cross-sectional studies have confirmed this association in childcare workers and teaching staff [26, 48, 49]. In contrast to the cross-sectional study [2], however, the association with the ERI model component OVC could not be confirmed in the follow-up. As a personality trait, OVC is a good predictor of burnout and this has been confirmed in a range of studies [27, 50–52]. The analysis also revealed that the burnout value for employees working in child care centres was around seven points higher on average than for employees from school partnership or youth organisations. ERI and burnout prevention measures should therefore be carried out, in particular, among employees working in child care centres.

### Limitations

One limitation of the study was the relatively small sample size. This resulted in wide confidence intervals and imprecise evaluations of the estimators. Furthermore, the relatively low follow-up rate resulted in a potential bias in the sample. A non-responder questionnaire was not carried out. On the basis of a dropout analysis, we attempted to identify potential selection effects and to take these into account.

Influential and outcome variables came from the same source – the presence of bias resulting from common methods, such as through social desirability, for example, could therefore not be excluded [53]. The factors of the ERI model only recorded part of the psychosocial situation in the workplace – no other psychosocial factors, such as those used in the job demand-control-support model [42], for example, were used – with the exception of control. Effects of a spill-over of psychosocial factors, but also biomechanical stress from employees’ private lives, also could not be excluded since these factors were not recorded as part of the study. Furthermore, part of the sample population (31%) took part in a parallel occupational preventive programme for the reduction of subjective noise exposure [28]. Although the study did not appear to have a statistically significant intervention effect, there were indications that the intervention group showed some benefits in terms of burnout as compared with the reference group. This subgroup was tested in the analyses of MS and burnout, but this characteristic was not shown to have any statistically significant influence. Despite this, it cannot be ruled out that the intervention may have had an effect on the individual level.

Over time, this study shows high and rising rates of burnout and ERI. As mentioned above, we cannot rule out that the professional-policy environment may have resulted in a classification bias of ERI, burnout and MS at the time that the data was collected. It is highly feasible that the protest movement by childcare workers in Germany at the time that the data was collected had sensitised the study participants and affected their responses.

For the high-risk group identified in the ERI model, those who showed an increased ERI and increased OVC were not shown to have an increased health risk in our study with regard to the outcome variables tested. Taking into account the study limitations, however, childcare workers with an effort-reward imbalance at baseline were shown to have an increased health risk with regard to MS and burnout at follow up.

The small sample size of childcare workers in Hamburg may not be representative for Germany, nevertheless, in comparison to a representative study of German childcare workers [3] there were no differences with respect to age, gender and nationality.

### Strengths

The main strength of this study was its longitudinal design. The analyses referred to prevalence rates at time of follow up and controlled for the outcome at baseline. The interpretation of the relation between independent and dependent variable was based on the chronology of time. Another strength, while investigating the relation between psychosocial factors and MS, was the assessment of physical stress and controlling for it in the models. By this approach we controlled a potential confounding effect of physical stress on the association of ERI and MS. Furthermore the assessment of psychosocial factors was performed with a validated instrument which was developed on the basis of a theoretical work stress model. With this approach the development of preventive measures is predetermined by the theory of the ERI model.

## Conclusions

As part of an occupational risk assessment, childcare workers were identified as an occupational group with a high ERI prevalence. In this context ERI was identified as a risk factor with regard to burnout and MS as part of a longitudinal approach. Measures should be developed at company level that can help to counter the increase of an effort-reward imbalance. Since monetary changes are hard to carry out at the company level, other measures should be implemented at this level to promote the sense of reward and decrease efforts. These may include the development of a culture that values and recognises its staff, which can be initiated at the management level. There are already empirical indications about the feasibility and success of ERI model based interventions aiming at a positive psychosocial work environment.

## Additional file

Additional file 1: Supporting information: Statement Personal Burnout COPSOQ Database. (PDF 97 kb)

## Abbreviations

BMI: Body mass index
ERI: Effort-reward imbalance
MS: Musculoskeletal symptoms
OR: Odds ratio
OVC: Overcommitment
